# Survival expectations in melanoma patients: a molecular prognostic model associated with aging

**DOI:** 10.1007/s12672-025-01971-z

**Published:** 2025-02-28

**Authors:** Nenghua Zhang, Xinyi Qiu, Xingying Chen, Cheng Du, Jingyi Dong, Xiaohong Li, Bing Chen, Lin Zhang, Yuyan Zhang

**Affiliations:** 1 Clinical Laboratory, Jiaxing Hospital of Traditional Chinese Medicine, Zhejiang Chinese Medical University, Jiaxing, 314033 China; 2 The First School of Clinical Medicine, The First Affiliated Hospital of Zhejiang Chinese Medical University, Hangzhou, 310053 China; 3 School of Life Sciences, Zhejiang Chinese Medical University, Hangzhou, 310053 China; 4 School of Pharmaceutical Sciences, Zhejiang Chinese Medical University, Hangzhou, 310053 China; 5 Ophthalmology Department, Jiaxing Hospital of Traditional Chinese Medicine, Zhejiang Chinese Medical University, Jiaxing, 314033 China

**Keywords:** Skin cutaneous melanoma, lncRNA, Aging, Risk model, Nomogram

## Abstract

**Background:**

Aging and long non-coding RNAs (lncRNAs) are research hotspots in melanoma. However, no study has so far explored the relationship between melanoma prognosis and aging-related lncRNAs (ARLs).

**Methods:**

The Cancer Genome Atlas database, the GTEx database, and the HAGR database were used in this study in a combined manner. Univariate and multivariate cox regression analyses were used to screen out lncRNA signatures associated with overall survival (OS) in the primary dataset. The risk scoring model was analyzed by risk stratification and tested internally. The protein expression levels of possible target genes of ARLs were verified by immunohistochemistry analysis in HPA database. Finally, gene enrichment analysis was performed.

**Results:**

In the primary dataset, five OS-related lncRNA signatures (AC011481.1, USP30-AS1, EBLN3P, LINC01527, HLA-DQB1-AS1) were screened out. The survival curve showed that the high-risk group had a worse prognosis than the low-risk group. The immunohistochemical analysis revealed that reduced expression of Epidermal Growth Factor Receptor (EGFR), along with increased expression of Activating Transcription Factor 2 (ATF2) and DNA Polymerase Delta 1 (POLD1), was linked to a worse prognosis. Finally, enrichment analysis revealed that OS-related DELs were significantly enriched in the regulation of reactive oxygen metabolism, etc. The ARGs were significantly activated in the SKCM tissues. The regulation of aging in melanoma cells may be realized through ferroptosis, immunity, and autophagy and so on.

**Conclusion:**

The ARL signature obtained in this study had better prognostic ability than individual clinical features.

**Supplementary Information:**

The online version contains supplementary material available at 10.1007/s12672-025-01971-z.

## Introduction

Skin cutaneous melanoma (SKCM) is a malignant tumor caused by the abnormal proliferation of melanocytes. Melanoma results in the death of 55,500 people annually, and it accounts for 75% of the fatalities related to skin diseases worldwide [1]. Owing to its high proliferation and metastatic potential, melanoma has become one of the main threats to human health and well-being [2]. Conventional radiotherapy has little effect on melanoma as most patients do not respond well [3]. In addition to early diagnosis and surgical resection of malignant melanoma, therapies such as immunotherapy and targeted therapy are available. However, immunoagent therapies do not work in more than half of the cases [4], and more than half of the people have tolerance to targeted therapeutic agents [5]. As a result, there is still a lack of specific therapies for the treatment of cutaneous melanoma. The 5-year overall survival rate of patients is only 30–40% [6]. Bioinformatics methods play a pivotal role in the discovery and study of predictive melanoma biomarkers, which facilitate enhanced diagnostic accuracy, treatment efficacy, and quality of life for melanoma patients [7]. Therefore, it is important to identify biomarkers that enable clinicians to accurately predict the prognosis of melanoma patients through bioinformatics methods, in conjunction with a substantial volume of sample data from public databases.

The mechanisms of cancer development involve the interplay of multiple factors, but it is often regarded as a degenerative disease related to aging, possibly linked to the decline in immune function and accumulated environmental factors. Both cancer and aging are caused by a time-dependent accumulation of damage in the cells [8]. Indeed, cancer and aging are two common features of the aging process [9]. A study has suggested that the aging microenvironment is strongly associated with tumor development and metastasis [10]. Biological aging often promotes the development, invasion, and metastasis of cancer, whereas cellular senescence serves as a potent tumor suppressor mechanism. The interaction between individual senescence and cell senescence is complex. A study by Dennis LK suggests that melanoma is associated with an increased cumulative lifetime exposure to sunlight in individuals with non-sun-sensitive skin (individuals with medium or dark skin tones), which may be direct evidence of the association between melanoma and aging [11]. However, there is no current genetic evidence establishing a correlation between the two. Aging-related genes (ARGs) play a vital role in cell senescence and senescence microenvironment, therefore, also function in tumor development and prognosis [12]. Recently the use of ARGs as a molecular biomarker for the diagnosis or prognosis of various types of cancer, such as colorectal cancer and lung adenocarcinoma, has attracted much attention [12, 13]. However, the biological function of ARGs and their potential prognostic role as a biomarker in SKCM are yet to be elucidated.

Long non-coding RNA (lncRNA) has almost no ability to encode proteins but is involved in epigenetic regulation [14]. A genome-wide association study of tumor samples has identified several cancer-related lncRNAs whose change of expression and mutation affect the development, metastasis, and prognosis of tumor [15]. Therefore, the identification of specific lncRNAs plays a pertinent role in cancer treatment and prognostic analysis. However, there are no relevant reports to prove the effect and molecular mechanism of aging-related lncRNAs (ARLs) on the prognosis of patients with melanoma.

In general, multi-gene models have higher predictive capabilities than single-gene models. Therefore, in this study, The Cancer Genome Atlas (TCGA) database was combined with the Genotype-Tissue Expression (GTEX) database and the Human Aging Genome Resource (HAGR) database to identify the lncRNA that are simultaneously associated with aging and the prognosis of melanoma. Furthermore, Cox regression analysis was performed to determine the ARL signature. An accurate pathological nomogram was constructed in combination with clinical features.

## Materials and methods

### Data acquisition and preparation

With reference to the TCGA database (https://www.cancergenome.nih.gov/), relevant clinical information and FPKM data of 471 melanoma samples and 1 normal sample were obtained. The RNA-seq datasets and the related clinical information of 324 normal skin samples exposed to sunlight were obtained from the GTEX database. However, given the small number of normal samples in TCGA, correction for batch effects in the two databases may erase their biological differences. We gave up batch effect rectification but used the "limma" package to normalize the raw data obtained from different databases. The Human Aging Genome Resource is an online dataset employed to study the biology of human aging. In total, 307 human ARGs were determined from HAGR database [16].

### Co-expression analysis and expression visualization of ARLs

ARGs of melanoma patients with the expression data were extracted from the TCGA database. Subsequently, the ARGs and lncRNA co-expression analyses of melanomas were assessed by using the limma package (version 4.1.0) of R software. ARLs were obtained using $p<0.001$ and $\left|cor\right|>0.45$ as the filter [17]. The “pheatmap” packages were used to visualize the ARGs and ARLs by heatmaps.

### Determination of OS-related lncRNAs in SKCM patients

After excluding patients with unknown survival time and other clinical information, we obtained 454 SKCM patients as the “entire dataset”. The primary dataset for the preliminary screening was composed of 50% randomly selected samples. First, we performed univariate Cox analyses and Kaplan–Meier analysis to evaluate the correlation between the expression of ARLs and OS ($p<0.05$). Meanwhile, we use three machine learning methods, namely, Random Forest, Support Vector Machine Regression and Elasticity Network, to identify the main features of ARLs. COX regression models provide clear explanations of variables which is particularly important in clinical studies. We chose further multivariate Cox regression analyses to construct prognostic models and calculated Akaike Information Criterion (AIC) values for the multifactorial models. Finally, we selected the model with the lowest AIC as the best-fitting predictive model.

### Identification and evaluation of ARL signature

After multivariate Cox regression analyses, we obtained the OS-related lncRNAs and their regression coefficients. Accordingly, the expression value of the related lncRNA and its regression coefficient could be linearly weighted. The risk scoring formula was constructed using the following formula:

$$Risk Score={\sum }_{i=1}^{n}\left({C}_{i}\times {Exp}_{i}\right)$$

where, $n$ is the number of screened lncRNA, ${Exp}_{i}$ is the expression value, and ${C}_{i}$ is the corresponding regression coefficient. This formula calculates the overall score by assigning specific weights to each variable, with these weights reflecting the strength of the association between each lncRNA and the risk of eventual outcome. In order to distinguish the patients’ prognostic status, we assigned the patients to two groups of high and low risks using the median risk score in the primary data set as the threshold. Kaplan–Meier survival curves were plotted for the two groups, respectively, and the log-rank method was employed to test for the presence of significant survival differences between the two groups. Finally, the “survival” package and “pheatmap” packages were used to plot the survival curves of the two groups and the heatmaps of screened lncRNA for visual display. To further evaluate the predictive performance of risk score, the time-dependent receiver operating characteristic (ROC) curves of the two datasets were plotted and the value of Area Under Curve (AUC) were calculated. One, three, five, and ten years were defined as the time nodes.

### Risk stratification analysis

Considering the high number of patients with SKCM and the presence of multiple confounding factors, a risk stratification analysis was carried out. The main confounding factors included age, T, and M stage. Therefore, 454 patients with SKCM were divided into two groups according to their age (< 60 or ≥ 60), T stage (T0-T2 subgroup and T3-T4 subgroup), or M stage (M0 subgroup and M1 subgroup).

### Establishment of a prognostic nomogram based on lncRNA signature

First, we employed the “survival” package to perform univariate and multivariate Cox analyses on the clinical factors of the patient and plotted the corresponding forest diagram. Overall survival was used as the prognostic index. As with the statistical standard, the clinical influencing factors exerting a significant effect on the prognosis were screened out. Then, ROC curve of clinical information and risk score were plotted to evaluate the prognostic value. To better predict the prognosis of patients, we finally used the “rms” package to build a prognostic nomogram combined with risk scores and clinical factors. The c-index and calibration curve were employed to evaluate the predictive effect of the nomogram.Identification and immunohistochemical validation of co-expressed mRNA.

The ARLs-ARGs coexpression network was visualized by using the Cytoscape software.

($|cor| > 0.3,p < 0.001$). The mRNA with the highest lncRNA signature correlation was screened out, and the Human Protein Atlas (HPA) database was used to verify its protein expression based on immunohistochemistry.

### Gene enrichment analysis

The targeted mRNA of ARLs were analysed using Gene Ontology (GO) and pathway enrichment analysis (Kyoto Encyclopedia of Genes and Genomes, KEGG). The mRNAs with the highest correlation were analysed using GSEA enrichment analysis (GSEA). The “clusterprofiler” R package were used to perform the enrichment analyses, respectively $p < 0.05$ was set as the statistical standard. Finally, the “Enrich plot” package was used to visualize the enrichment outcomes.

### Statistical analysis

Data processing was performed using the Perl programming language and R software (version 4.2.1). Different statistical analysis processes have different thresholds for statistical significance. The specific p-value was expounded in the procedure mentioned earlier. Statistical analysis of baseline information selected the student’s t-test. Two-tailed P-values were reported.

## Results

### Co-expression analysis and expression visualization of ARLs

**Fig. 1:**
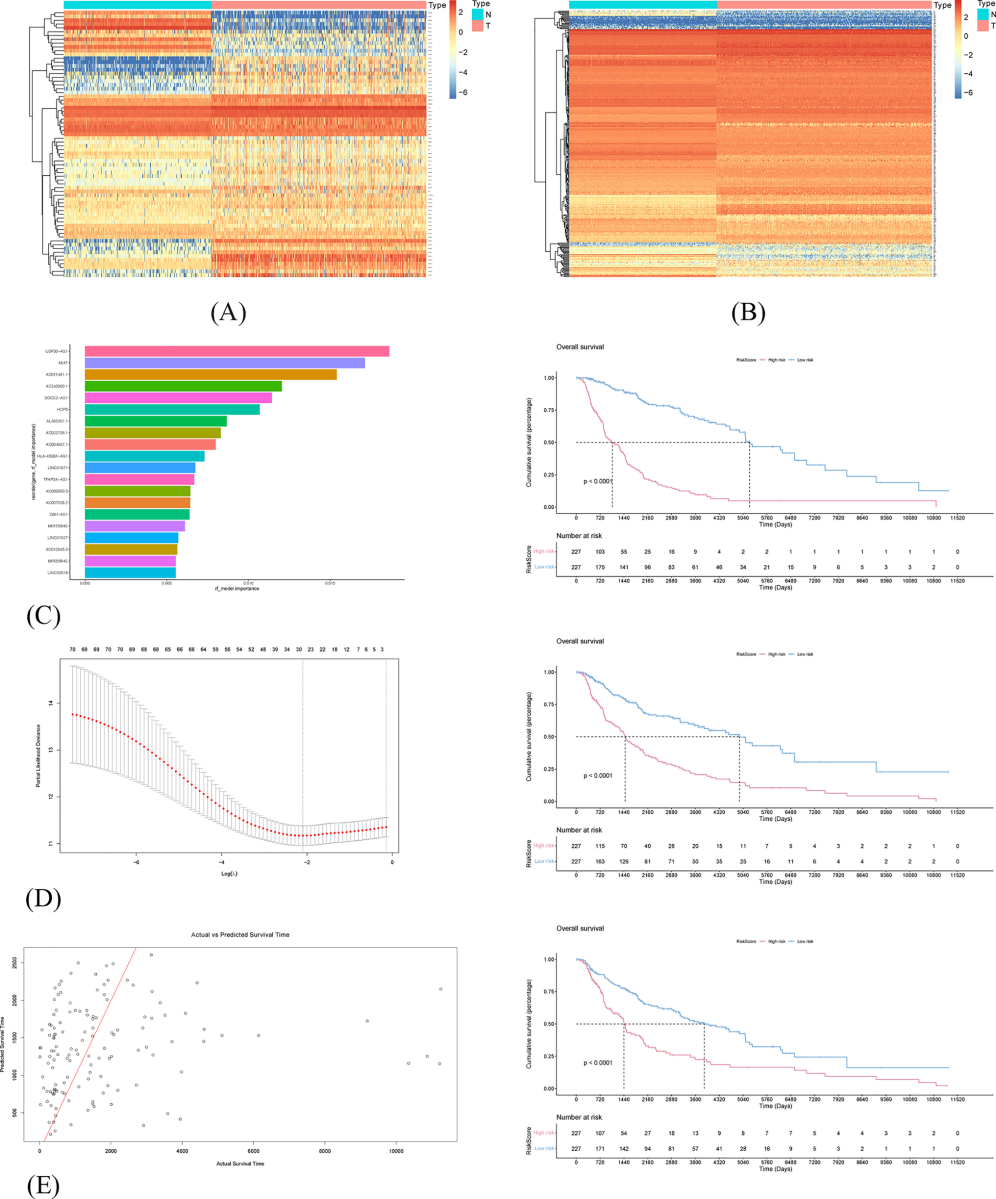
Screening and expression visualization of ARLs based on machine learning algorithms. **A** The heatmap of 71 ARLs. **B** The heatmap of 307 ARGs. **C** Feature importance assessment plot and survival curve based on random forest model. **D** Parameter λ Optimal value selection process and survival curves for elastic network

307 ARGs were downloaded from the HAGR database (Table S1). The intersection of ARGs and TCGA-mRNAs was taken to obtain the 302 ARGs and their expression matrices. Using $p<0.001$ and $\left|cor\right|>0.6$ as the filter, 71 co-expressed ARLs were screened. The gene expression heatmaps of ARLs and ARGs showed significant differences between normal and tumor samples (Fig. 1A–B).

### Determination of the OS-related ARLs

**Fig. 2:**
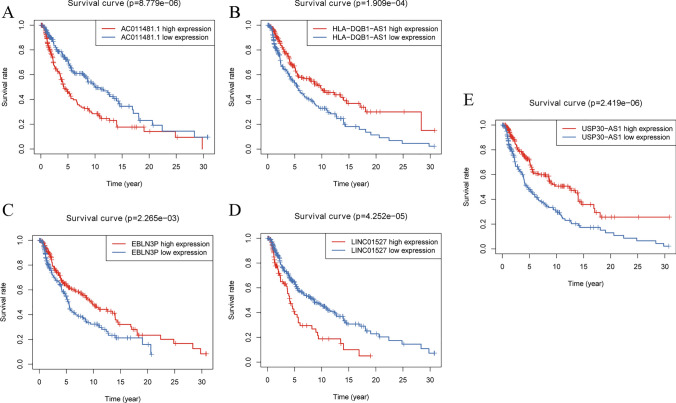
Survival curves of 5 OS-related ARLs in the entire dataset (all p < 0.01)

The baseline characteristics of the entire dataset and the primary dataset were shown in Table 1. The clinical information of 228 patients in the primary dataset were shown in Table S2. Firstly, in the primary dataset, eighteen lncRNAs significantly associated with OS were screened out ($p < 0.01$), as shown in Table 2. The most important feature ARL identified by Random Forest was USP30-AS1, and the top 20 ARLs in terms of importance are shown in Fig. 1C. The optimal value of the parameter λ was 0.079 and the α was 0.4 for the resilient network regression explicitly (Fig. 1D). Scatterplots were used to visualize the prediction results of the support vector machine (Fig. 1E). The deployment of three distinct machine learning algorithms has been instrumental in our endeavor to pinpoint lncRNAs that exhibit a significant correlation with OS. Survival analysis based on the entire dataset clarified that there was a significant difference between the high-risk and low-risk groups classified using all three machine learning algorithms. Subsequently, five OS-related ARLs with the highest likelihood ratio and the lowest AIC were selected by multivariate Cox regression analysis and AIC values, as shown in Table 3. Three lncRNAs (USP30-AS1, EBLN3P, and HLA-DQB1-AS1) that served as protective factors had hazard ratios (HR) < 1. On the contrary, AC011481.1 and LINC01527 were the risk factors (HR > 1). Subsequently, based on the entire dataset, the survival curves of these five genes were plotted for verification (Fig. 2).

**Table 1:** Clinical characteristics of 454 SKCM patients involved in this research

Clinical Characteristic	Primary dataset	Entire dataset	P-value
	n = 228	n = 454	
Age(year)			0.62
≥ 60	102	215	
< 60	126	239	
Gender			0.41
female	79	172	
male	149	282	
Stage			0.64
0–II	107	228	
III–IV	102	191	
unknown	19	35	
T			0.31
T0–T2	77	140	
T3–T4	116	236	
Tis	4	7	
unknown	31	71	
M			0.72
M1	13	23	
M0	201	405	
unknown	14	26	
N			0.79
N0	108	225	
N1–N3	93	176	
unknown	27	53

**Table 2:** Univariate Cox analyses and Kaplan–Meier survival analyses of OS-related DELs in the primary dataset

Gene	HR	HR.95L	HR.95H	*p* value
AC011481.1	1.71	1.20	2.43	3.02E-03
USP30-AS1	0.67	0.53	0.84	5.01E-04
AL512274.1	1.43	1.10	1.86	8.12E-03
AC018755.4	0.69	0.52	0.91	8.83E-03
AC124319.1	0.63	0.47	0.86	2.98E-03
AC011481.1	1.71	1.20	2.43	3.02E-03
EBLN3P	0.52	0.37	0.71	5.21E-05
LINC01871	0.77	0.65	0.91	2.68E-03
AL133371.2	0.61	0.44	0.85	2.90E-03
MIR155HG	0.72	0.58	0.90	3.72E-03
AC012236.1	0.64	0.48	0.87	4.17E-03
LINC02446	0.68	0.55	0.86	1.00E-03
PSMB8-AS1	0.73	0.61	0.88	9.05E-04
LINC01527	1.64	1.22	2.21	1.14E-03
HCP5	0.79	0.70	0.90	3.34E-04
MIAT	0.60	0.41	0.87	7.57E-03
AC243960.1	0.64	0.49	0.85	1.61E-03
HLA-DQB1-AS1	0.65	0.52	0.82	2.04E-04

**Table 3:** Multivariable Cox analyses of 5 OS-related DELs associated with aging in primary dataset

Gene	coefficient	HR	HR.95L	HR.95H
AC011481.1	0.30	1.34	0.92	1.96
USP30-AS1	−0.24	0.78	0.60	1.03
EBLN3P	−0.45	0.64	0.44	0.91
LINC01527	0.33	1.39	1.00	1.93
HLA-DQB1-AS1	−0.26	0.77	0.59	1.01

### Identification and evaluation of the risk prognosis models

According to the regression coefficients of five OS-related ARLs, the risk scoring model was obtained by linear weighting as following:

$$Risk Score=0.30\times {Exp}_{AC011481.1}-0.24\times {Exp}_{USP30-AS1}-0.45\times {Exp}_{EBLN3P}+0.33\times {Exp}_{LINC01527}-0.26\times {Exp}_{HLA-DQB1-AS1}$$

**Fig. 3:**
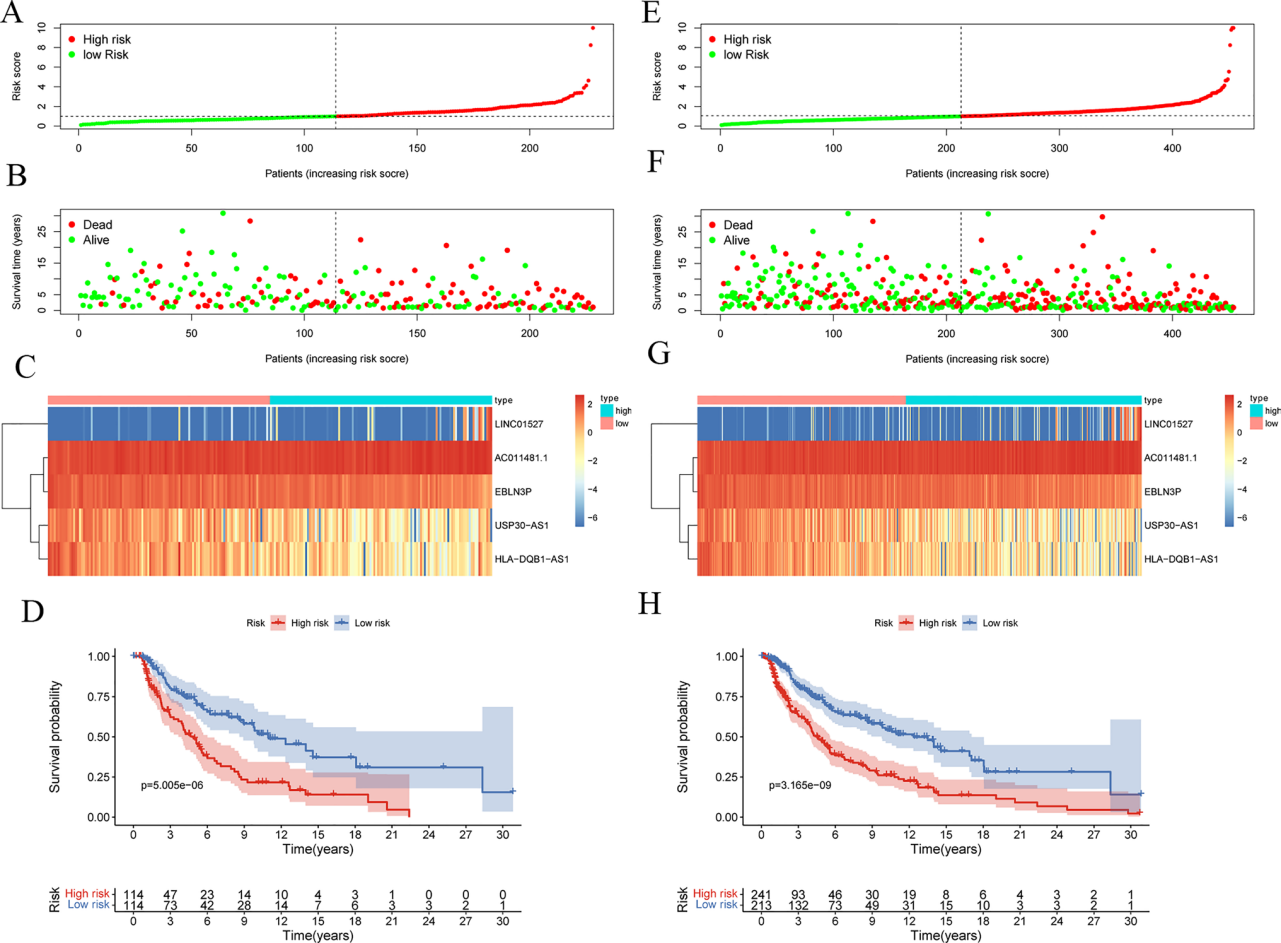
Visualization and model testing of risk prognosis models results in primary dataset and entire dataset. **A** The risk score grouping in primary dataset. **B** OS state scatters dia-gram in primary dataset. **C** heatmap of the aging-related lncRNA signature in primary dataset. **D** The survival curves in primary dataset. Red and blue represent the high- and low-risk groups, respectively. **E** The risk score grouping in entire dataset. **F** OS state scatters diagram in entire dataset. **G** heatmap of the aging-related lncRNA signature in entire dataset. **H** The survival curves in entire dataset

**Fig. 4:**
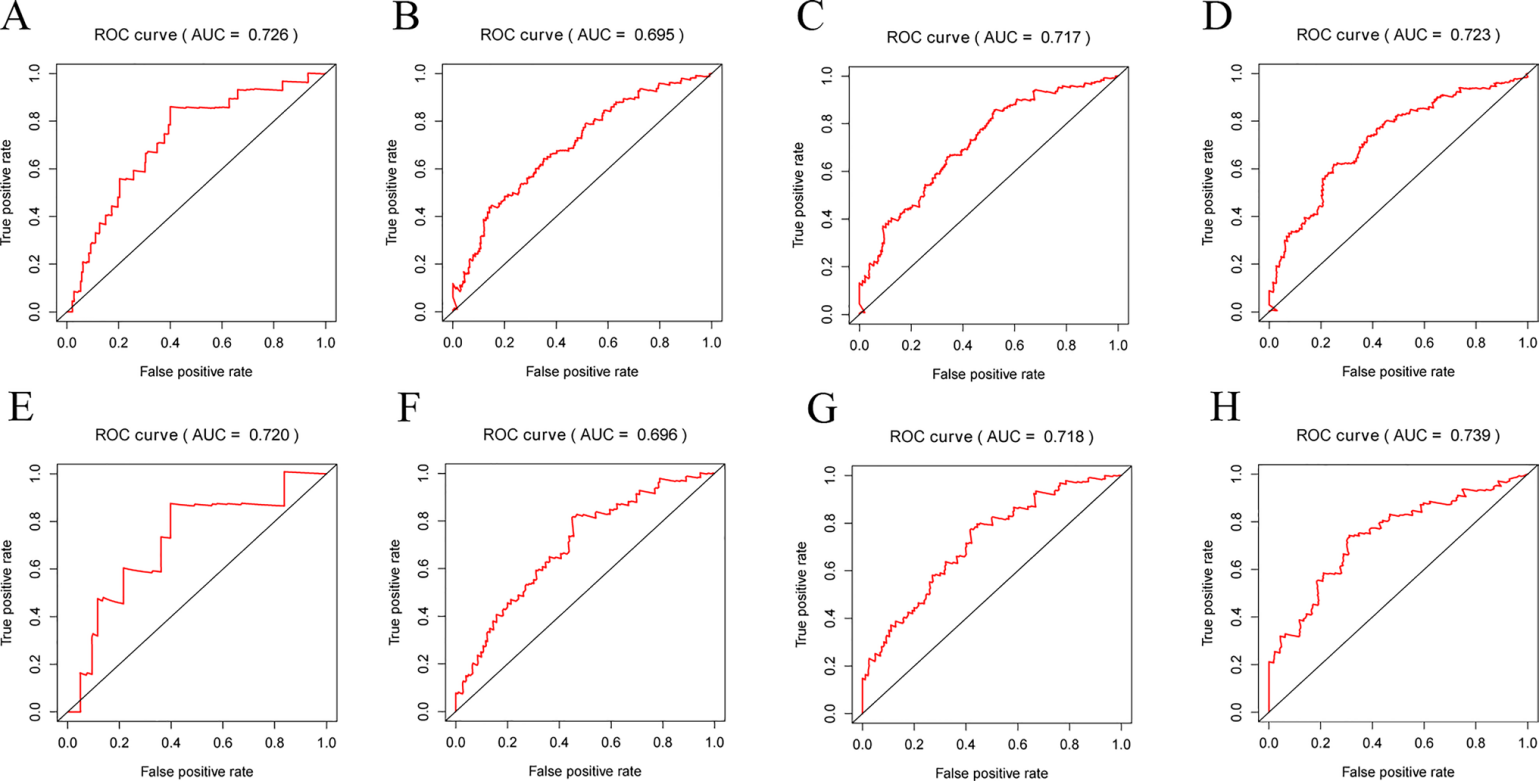
ROC curves at different time nodes. **A** ROC curve for the primary dataset at 1 year (AUC = 0.726). **B** ROC curve for the primary dataset at 3 years (AUC = 0.695). **C** ROC curve for the primary dataset at 5 years (AUC = 0.717). **D** ROC curve for the primary dataset at 10 years (AUC = 0.723). **E** ROC curve for the entire dataset at 1 year (AUC = 0.720). **F** ROC curve for the entire dataset at 3 years (AUC = 0.696). **G** ROC curve for the entire dataset at 5 years (AUC = 0.718). **H** ROC curve for the entire dataset at 10 years (AUC = 0.739)

In primary dataset, the grouping of the high-risk and low-risk groups is shown in Fig. 3A. The OS state scatters diagram (Fig. 3B) and heatmap of the aging-related lncRNA signature (Fig. 3C) showed that there were certain differences in the survival status between the two groups. The survival curve is depicted in Fig. 3D. The survival curve revealed the presence of statistically significant differences between the two groups (p = 2.696 E-06). In addition, the data distribution state of the entire dataset demonstrated the similar trend (Fig. 3E–H). Further, 1, 3, 5, and 10 years were selected as time nodes. ROC curves of the primary dataset (Fig. 4A–D) and the entire dataset (Fig. 4E–H) were plotted. In the primary dataset and the entire dataset, the AUC values for 1, 3, 5, and 10 years were 0.720, 0.696, 0.718, and 0.739 and 0.726, 0.695, 0.717, and 0.723, respectively. In general, the risk model exhibited good and stable classification performance.

### Risk stratification analysis

**Fig. 5:**
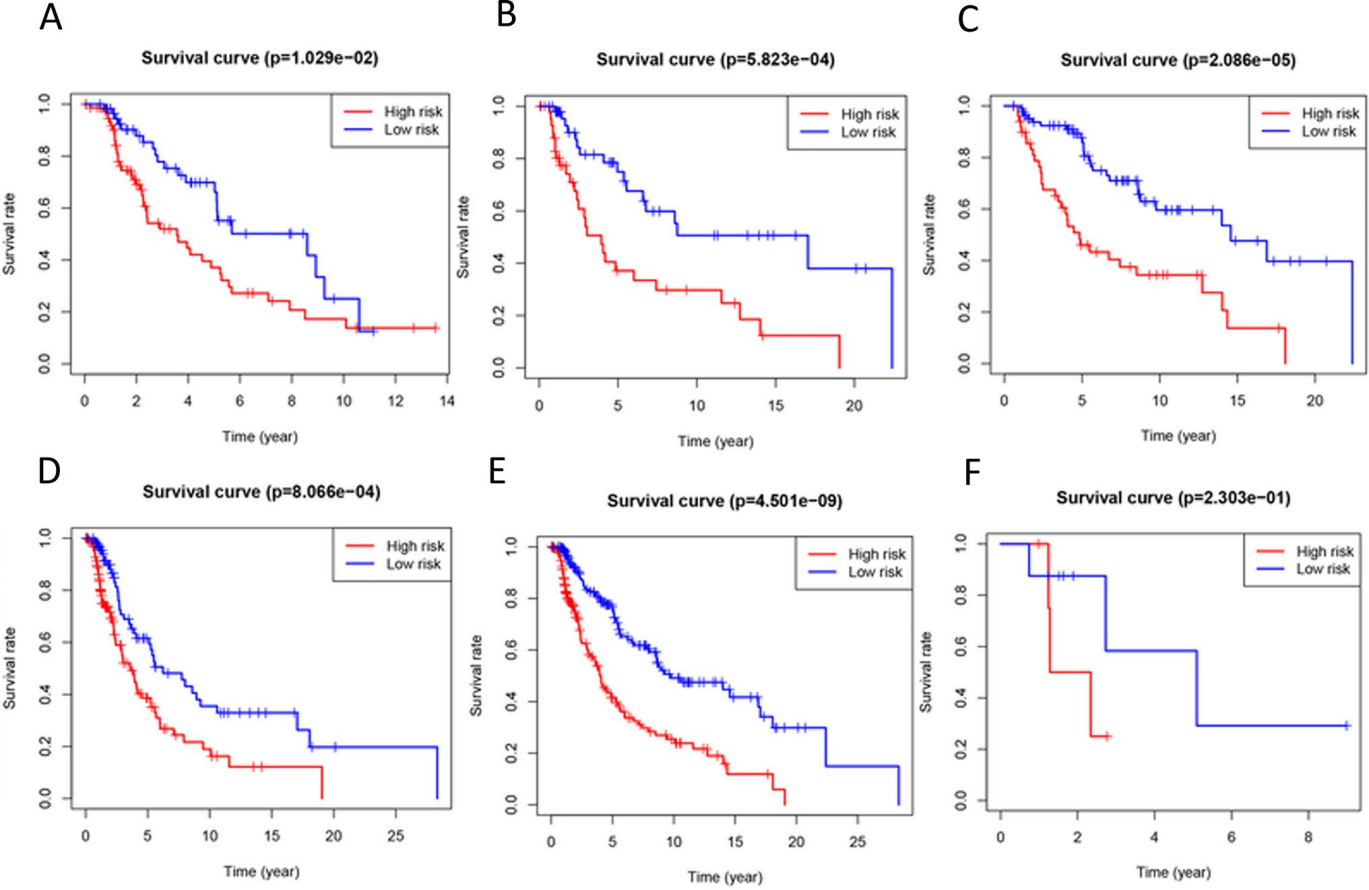
Risk stratification analysis based on the five-lncRNA signature. **A ** ≥ 60-year-old sub-group. **B**  < 60-year-old subgroup. **C** T0-T2 subgroup. **D** T3-T4 subgroup. **E** M0 subgroup. **F** M1 subgroup.

In this study, 454 patients with SKCM were divided into two groups according to their age (< 60 or ≥ 60). In the different age subgroups, there were significant differences between the high-risk and low-risk groups in the survival curves. Relatively, the effect of the age < 60 subgroup was more obvious than the age ≥ 60 subgroup (Fig. 5A–B). In the T0-T2 subgroup and T3-T4 subgroup, the five-lncRNA signature had significant and similar classification effects (Fig. 5C–D). However, in the hierarchical analysis based on the M-index, we found that the application of five-lncRNA signature to the M0 subgroup had a good classification effect, but the effect was poor in the M1 subgroup (Fig. 5E–F). This might be the result of the small sample size of patients with distal metastasis. In summary, the classification effect of five-lncRNA signature was excellent in most subgroups.

### Establishment and validation of nomogram

**Fig. 6:**
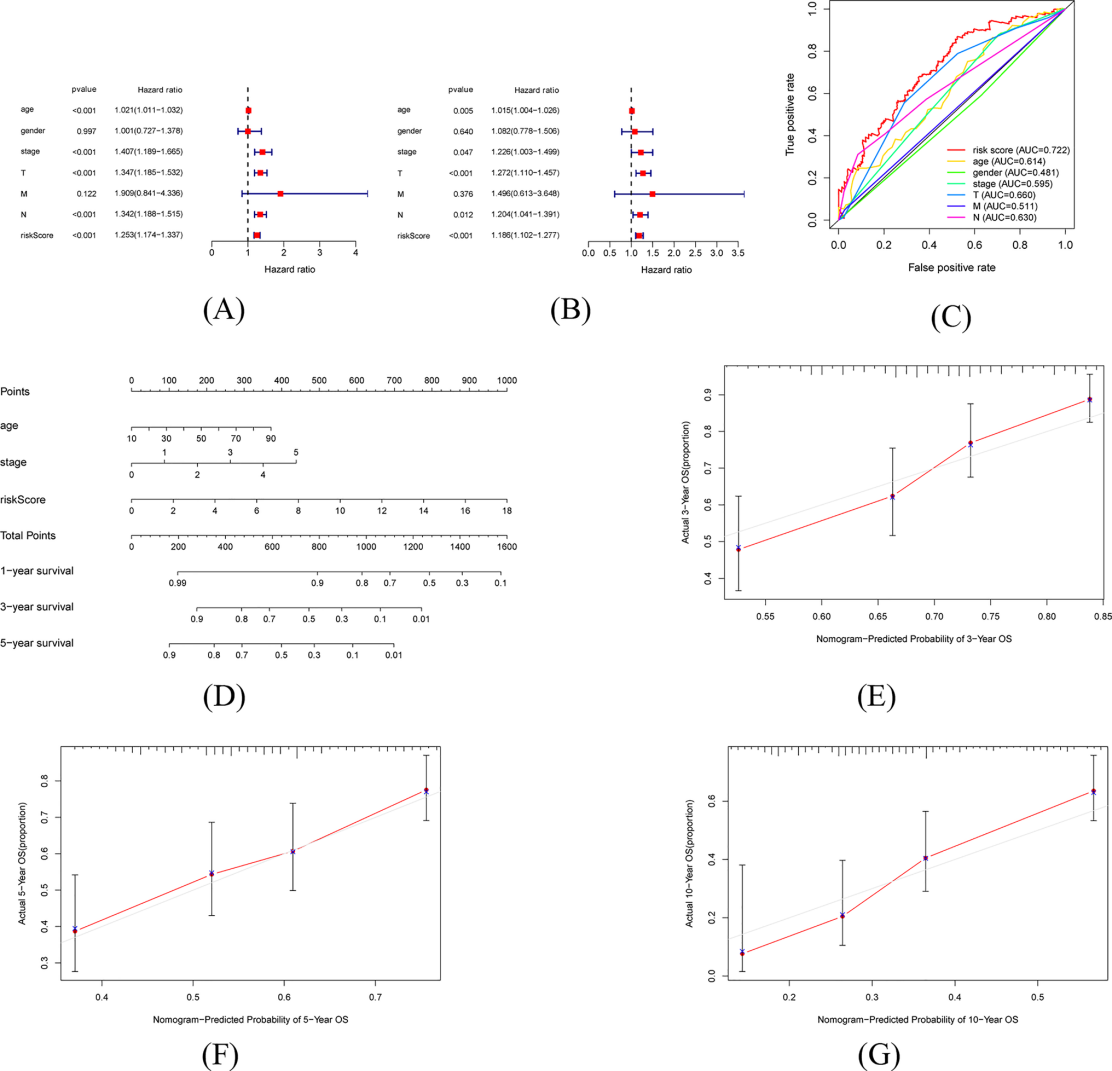
Screening for independent prognostic factors and establishment of nomograms. **A** Forest map showing independent prognostic clinical factors in univariate COX analyses. **B** Forest map showing independent prognostic clinical factors in multivariate Cox analysis. **C** ROC curves for independent factors. **D** Nomogram combining the clinical factors and risk scores. **E** Calibration curves of 3-year. **F** Calibration curves of 5-year. **G** Cali-bration curves of 10-year

With $p < 0.05$ as the statistical standard, univariate and multivariate Cox analyses showed that age, stage, T, N, and risk score can independently predict the prognosis of SKCM patients (Fig. 6A–B). To avoid multicollinearity, two clinical indicators of T, N were abandoned. Meanwhile, to evaluate thcce prediction accuracy of the signature, the ROC curves were plotted, and the AUC values were calculated in the entire dataset (Fig. 6C). By taking 5 years as the time node, the aging-related risk score was concluded to be the best predictor when compared with the other clinical factors. Then all the independent prognostic factors were recruited to construct the nomogram (Fig. 6D). Calibration curves and C-index were used to evaluate the predictive effectiveness of the nomograms. With the red line being close to the solid grey line, the calibration curves suggested that the prediction of survival probability of 3-, 5-, and 10-year by the nomogram agreed with the observed values (Fig. 6E–G). The C-index was 0.713 (95% CI 0.691–0.735) for the entire dataset.

### Co-expression networks and immunohistochemical validation

**Fig. 7:**
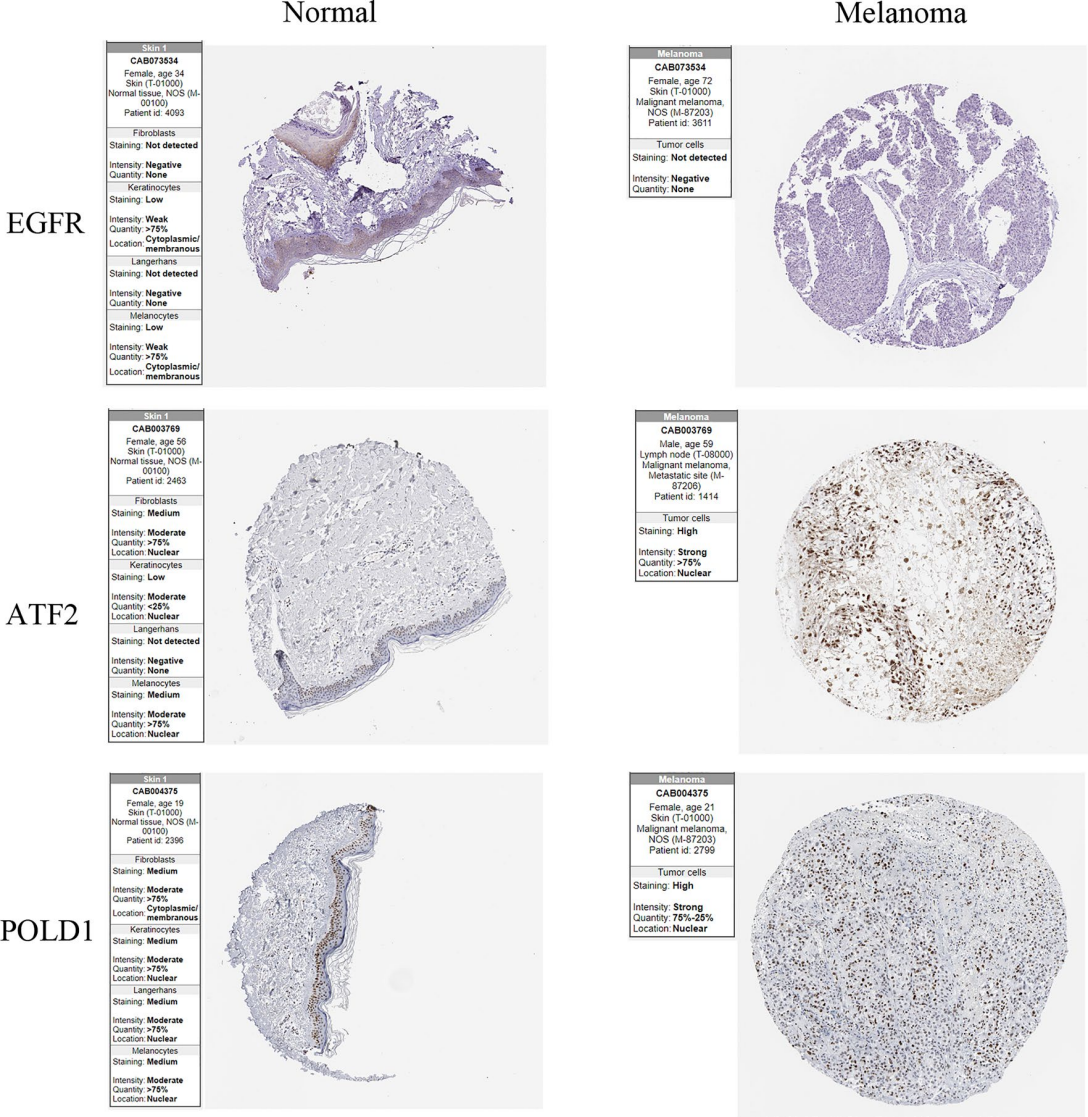
IHC of target genes in normal tissues and melanoma tissues from the HPA database

The co-expression network of 5 lncRNAs obtained by univariate Cox analysis was performed using the Cytoscape ($|cor| > 0.3,p < 0.001$) (Figure S1). ARLs-ARGs with the highest correlation is shown in Table 4. HPA database was used to verify the expression of mRNA based on immunohistochemistry (Fig. 7). Decreased EGFR expression and increased ATF2 and POLD1 expression were associated with poorer prognosis. The results are consistent with our study.

**Table 4:** ARLs and ARGs with the highest correlation

lncRNA	mRNA	cor	P-value
AC011481.1	POLD1	0.62	1.52E-51
EBLN3P	ATF2	0.65	7.24E-57
HLA-DQB1-AS1	IL2RG	0.64	4.65E-56
LINC01527	TP63	0.68	2.18E-66
USP30-AS1	IL2RG	0.87	2.65E-145

### Gene set enrichment analysis

**Fig. 8:**
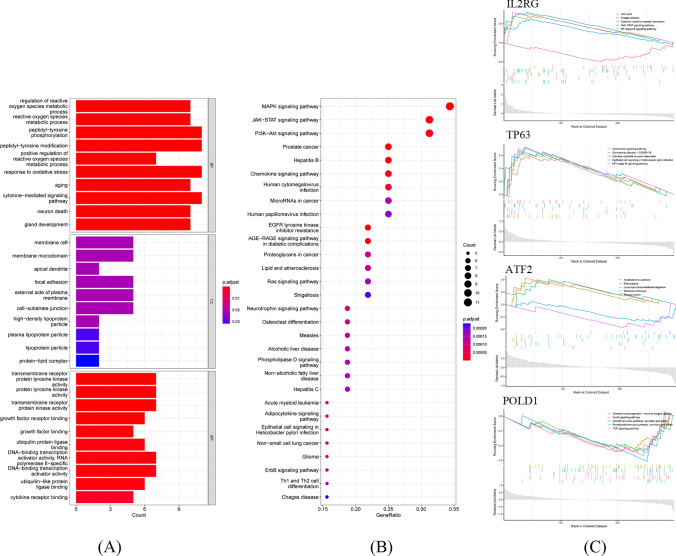
Functional enrichment of the ARGs. **A** Bar graph of the GO enrichment results. **B** The KEGG enrichment results were presented in the bubble plot. **C** Gene Set Enrichment Analysis of highest correlation ARGs

The GO results showed that the screened ARGs were mainly enriched in the regulation of reactive oxygen species (ROS) metabolism, neuron death, and aging (Fig. 8A). The KEGG enrichment results revealed that ARGs were chiefly involved in MAPK, JAK − STAT, and PI3K–Akt signaling pathways (Fig. 8B). The ARGs were significantly activated in the SKCM tissues. GSEA enrichment analysis of ARGs with the highest correlation with ARLs demonstrated the differences in enriched pathways between the high-expression and low-expression groups, specifically displaying the top five significantly enriched pathways (Fig. 8C).

## Discussion

The aging microenvironment has been shown to be closely related to the metastasis and therapeutic resistance of melanoma [18]. However, our current approaches in the development of biomarkers for melanoma are focused on fluid markers, immune-related markers (Table 5) [19, 20]. There have been no studies on the prognostic value of ARLs in melanoma. Owing to the complexity of the aging process, this study attempted to explore the specific biological process and the related molecular mechanism via the analysis of the ARL expression pattern. The effect of ARLs on the prognosis of patients with SKCM was chiefly evaluated.

**Table 5:** Immune-related biomarkers and liquid biopsy markers of melanoma

Type	Biomarker	Research
Immune-related markers	Protein levels (GPER1 and COL17)	Uğur Çakı, etc.[21]
	Protein levels (CRABP2)	Shuangshuang Zeng, etc.[22]
	Gene levels (MUC16 and TTN)	Nilesh Kodal, etc.[23]
	Gene levels (DSC2 and DSC3)	Dongyun Rong, etc.[24]
Liquid biopsy	CTCs, ctDNA and miRNAs	Nicholas Slusher, etc.[25]
	CNV	E Lukacova, etc.[26]
	CCL20	Julian Kött, etc.[27]
	EV	Mickensone Andre, etc.[28]

*GPER1* G-protein coupled estrogen receptor 1, *COL17* endodomain of collagen XVII, *CRABP2* Cellular retinoic acid-binding protein 2, *CTCs* circulating tumor cells, *ctDNA* circulating tumor DNA, *miRNAs* circulation microRNAs, *CNV* copy number variations, *CCL20* C–C motif chemokine ligand 20, *EV* extracellular vesicles

In our study, an aging-related signature comprising five ARLs was developed, including AC011481.1, USP30-AS1, EBLN3P, LINC01527, and HLA-DQB1-AS1. After ROC curve validation and risk stratification analysis, aging-related risk scores were identified to be the stable independent prognostic factors. The risk model exhibited good and stable classification performance in both primary dataset and entire dataset. Based on the findings, GO and KEGG enrichment analyses were subsequently conducted.

Interleukin 2 receptor subunit Gamma chain(IL2Rg) is a common receptor subunit of many important immune factors, which play a unique role in the immune system [29]. IL2Rg gene knockout is often used to construct immunodeficient mice to verify the efficacy of immunotherapy. Previous studies have suggested that mutations in IL2RG gene may be associated with virus-derived skin infections [30]. The number of tumor-causing melanoma cells was significantly increased in Il2rg(-/-) mice with interleukin-2 receptor γ -chain deletion [31]. Both the screened lncRNA USP30-AS1 and HLA-DQB1-AS1 showed a high correlation with IL2RG, suggesting the association between aging and immunity. IL2Rg may also be an important immunotherapy target for melanoma. Meanwhile, USP30-AS1, LINC01527 and HLA-DQB1-AS1 were also considered to be an independent prognostic marker of melanoma as an autophagy-related lncRNA [17, 32]. In addition, related studies have observed that USP30-AS1 played a role as a biomarker in the prognostic prediction of bladder and urethral carcinoma, ovarian cancer, and cervical cancer [33–35]. Interestingly, USP has been screened out with multivariate Cox analysis as a protective factor in melanoma (HR < 1). However, in acute myeloid leukemia (AML), USP30-AS1 appears to be a risk factor for inducing immune escape of the AML cells [36]. Considerable studies have confirmed that EBLN3P can promote the progression of liver cancer, osteosarcoma and colorectal cancer [37–39]. However, the role of EBLN3P in SKCM has not been confirmed by relevant experiments. AC011481.1 is yet another independent prognostic signature of melanoma screened out using multivariate Cox analysis. No relevant studies have been performed on it. However, the DNA polymerase δ 1 catalytic subunit (POLD1) co-expressed with AC245041.1 was found to be associated with the prognosis of liver cancer, colorectal cancer, and endometrial cancer [40, 41]. At the same time, clinically, patients with POL /POLD1 mutations are often accompanied by skin pigmentation, pilomatricoma and other skin lesions [42, 43]. However, the mechanism of POLD1 action in melanoma has not been explored.

Moreover, TP63, the mRNA coexpressed with LINC01527, has been found to be an effective biomarker in the diagnosis of SKCM. P63 is associated with many biological processes in the cells, especially epithelial biology [44]. P63 was observed to be significantly overexpressed in cutaneous basal cell carcinoma (cBCC), another type of skin cancer with a better prognosis than melanoma [45]. It is generally believed that the phenomenon of P63 overexpression is not significant in melanoma cells, but some studies have found up to 60% p63 positivity in a batch of melanoma samples. This finding demonstrates that TP63 has an antiapoptotic effect in melanoma and is responsible for mediating chemoresistance [46]. In our study, LINC01527 was identified to be a risk factor positively correlated with the expression of TP63, which is consistent with previous research [46]. To further explore the mechanism, we have compiled and organized the relevant literature for ARL and ARG, as shown in Table 6.

**Table 6:** Research on Related Functions and Mechanisms of LncRNA and mRNA

LncRNA	Research on Related Functions and Mechanisms	mRNA	Research on Related Functions and Mechanisms
AC011481.1	**/**	POLD1	Related to the high microsatellite instability and tumor immunotherapy[47]
EBLN3P	Promoting the progression and metastasis of liver cancer, osteosarcoma, and colorectal cancer[38, 48, 49]	ATF2	Promoting the progression of melanoma and renal cell carcinoma[50] [51]; Immunomodulatory effects[52]
HLA-DQB1-AS1	Necroptosis[53]; autophagy-related[54]; immune-related[55]	IL2RG	Immunomodulatory and Immunodeficiency relatedness[56] [57]; Renal cell carcinoma prognosis related[58]
LINC01527	autophagy-related[54]	TP63	Squamous cell carcinoma adn prostate cancer-specific oncogene[59] [60]; Promotion of pyroptosis[61]
USP30-AS1	Immunomodulation of viral infections[62]; Cancer prognosis-related lncRNA studies. (Adenocarcinoma of the colon [9], cervical cancer[63], breast cancer[64], ovarian cancer[65], bladder cancer[66], melanoma[17, 67, 68]	IL2RG	**/**

The GO biological process category indicated that the main enrichment is in aging, ROS metabolism, and regulation of cell adhesion. In fact, considering that lipids are important targets of ROS, the ROS metabolism level reflects the lipid peroxidation level in the mitochondria to some extent [69]. At the same time, the accumulation of lipid-based ROS in the melanoma cells causes ferroptosis. During this process, the targeting effect of miR-137 on the glutamine transporter SLC1A5 in the melanoma cells plays a negative role in regulating ferroptosis [70]. It is speculated that ARL might also have a similar mechanism that affects tumor growth by regulating the ferroptosis process in the cells.

The enrichment results of KEGG were similar to those of GO. The genes were mainly enriched in MAPK, PI3K − Akt, JAK − STAT signaling pathways and so on. In fact, these three pathways are closely related to each other, and there has been considerable research on them. For example, computational models of PI3K/Akt and MAPK signaling pathways in melanoma have been established [71]. The JAK − STAT signaling pathway can transmit signals to the PI3K − Akt signaling pathway to further activate downstream pathways, such as protein translation, apoptosis, and P53 pathway, and further regulate tumor development and metastasis. MERTK receptor tyrosine kinase has been found to be an effective therapeutic target for melanoma, regulating MAPK/ERK, PI3K/Akt, and JAK/STAT pathways simultaneously [72]. In recent years, the widespread application of MAPK-targeted therapy has significantly improved the OS in patients with advanced melanoma [73, 74]. However, the viewpoint that these three pathways are related to the aging process has not been proposed.

The limitation of this study is the potential bias caused by many confounding factors. However, the database used in this study is relatively comprehensive. At the same time, we conducted risk stratification analysis for some confounding factors to minimize the impact of bias on the results. Overall, considering the correlation between aging and the prognosis of patients with melanoma, five OS-related lncRNAs were identified through coexpression analysis of ARGs and lncRNAs as well as univariate and multivariate Cox regression analysis of ARLs. Next, the ARL signature was established. Finally, the clinical indicators and risk scores were combined to establish nomograms related to the aging process to help clinicians with the decision-making process.

## Conclusion

**Fig. 9:**
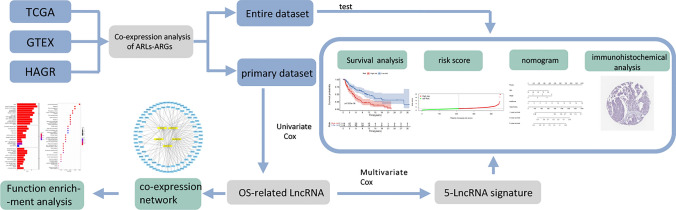
We harnessed the collective power of The Cancer Genome Atlas, GTEx, and HAGR databases to conduct a comprehensive analysis. We employed univariate and multivariate Cox regression analyses to meticulously identify lncRNA signatures that are significantly correlated with overall survival (OS) within our primary dataset. Subsequently, we developed a risk scoring model, which was rigorously evaluated through risk stratification and internal validation. To further substantiate our findings, we utilized immunohistochemistry analysis within the Human Protein Atlas (HPA) database to confirm the protein expression levels of potential target genes associated with ARLs. Concluding our investigation, we conducted a gene enrichment analysis to elucidate the biological significance of our discoveries. Our primary dataset yielded five lncRNA signatures that were found to be significantly associated with overall survival

In this study, TCGA, GTEx, and HAGR databases were used in a combined manner(Fig. 9). Importantly, the ARL signature was constructed, and the possibility of it functioning as an independent prognostic biomarker was verified. Finally, a clinical nomogram related to the aging process was established to augment our understanding of the prognosis of SKCM. Nonetheless, further investigations are needed to confirm the present findings.

## Supplementary Information

Additional file1 (DOCX 2025 KB)

## Data Availability

The datasets generated during and/or analysed during the current study are available in the [The Cancer Genome Atlas Program; GTEx database and the Human Ageing Genomic Resources database] repository, [https://www.cancer.gov/ccg/research/genome-sequencing/tcga; https://www.gtexportal.org/home%5B33; https://www.genomics.senescence.info/].

## References

[1] Schadendorf D, van Akkooi ACJ, Berking C, Griewank KG, Gutzmer R, Hauschild A, Stang A, Roesch A, Ugurel S. Melanoma. Lancet. 2018;392(10151):971–84. 30238891 10.1016/S0140-6736(18)31559-9

[2] Wei CY, Zhu MX, Lu NH, Peng R, Yang X, Zhang PF, Wang L, Gu JY. Bioinformatics-based analysis reveals elevated MFSD12 as a key promoter of cell proliferation and a potential therapeutic target in melanoma. Oncogene. 2019;38(11):1876–91. 30385854 10.1038/s41388-018-0531-6 PMC6462865

[3] Stull CM, Clark D, Parker T, Idriss MH, Patel VA, Migden MR. Current and emerging intralesional immunotherapies in cutaneous oncology. J Am Acad Dermatol. 2024. 10.1016/j.jaad.2024.05.095. 38942173 10.1016/j.jaad.2024.05.095

[4] Franklin C, Mohr P, Bluhm L, Meier F, Garzarolli M, Weichenthal M, Kähler K, Grimmelmann I, Gutzmer R, Utikal J et al. Brain metastasis and survival outcomes after first-line therapy in metastatic melanoma: a multicenter DeCOG study on 1704 patients from the prospective skin cancer registry ADOREG. J Immunother Cancer. 2023;11:e005828. 10.1136/jitc-2022-005828 PMC10083858 37028819

[5] Long GV, Swetter SM, Menzies AM, Gershenwald JE, Scolyer RA. Cutaneous melanoma. Lancet. 2023;402(10400):485–502. 37499671 10.1016/S0140-6736(23)00821-8

[6] Gershenwald JE, Scolyer RA, Hess KR, Sondak VK, Long GV, Ross MI, Lazar AJ, Faries MB, Kirkwood JM, McArthur GA, et al. Melanoma staging: evidence-based changes in the American Joint Committee on Cancer eighth edition cancer staging manual. CA Cancer J Clin. 2017;67(6):472–92. 29028110 10.3322/caac.21409 PMC5978683

[7] Yang E, Ding Q, Fan X, Ye H, Xuan C, Zhao S, Ji Q, Yu W, Liu Y, Cao J, et al. Machine learning modeling and prognostic value analysis of invasion-related genes in cutaneous melanoma. Comput Biol Med. 2023;162:107089. 37267825 10.1016/j.compbiomed.2023.107089

[8] Aunan JR, Cho WC, Soreide K. The biology of aging and cancer: a brief overview of shared and divergent molecular hallmarks. Aging Dis. 2017;8(5):628–42. 28966806 10.14336/AD.2017.0103 PMC5614326

[9] Santos F, Moreira C, Nobrega-Pereira S. Bernardes de Jesus B: new insights into the role of epithelial(-)mesenchymal transition during aging. Int J Mol Sci. 2019. 10.3390/ijms20040891. 30791369 10.3390/ijms20040891 PMC6412502

[10] Fane M, Weeraratna AT. How the ageing microenvironment influences tumour progression. Nat Rev Cancer. 2020;20(2):89–106. 31836838 10.1038/s41568-019-0222-9 PMC7377404

[11] Dennis LK. Cumulative sun exposure and melanoma in a population-based case-control study: does sun sensitivity matter? Cancers (Basel). 2022. 10.3390/cancers14041008. 35205756 10.3390/cancers14041008 PMC8870683

[12] Xu Q, Chen Y. An aging-related gene signature-based model for risk stratification and prognosis prediction in lung adenocarcinoma. Front Cell Dev Biol. 2021;9:685379. 34277626 10.3389/fcell.2021.685379 PMC8283194

[13] Yue T, Chen S, Zhu J, Guo S, Huang Z, Wang P, Zuo S, Liu Y. The aging-related risk signature in colorectal cancer. Aging (Albany NY). 2021;13(5):7330–49. 33658390 10.18632/aging.202589 PMC7993742

[14] Zhao X, Li X, Zhou L, Ni J, Yan W, Ma R, Wu J, Feng J, Chen P. LncRNA HOXA11-AS drives cisplatin resistance of human LUAD cells via modulating miR-454-3p/Stat3. Cancer Sci. 2018;109(10):3068–79. 30099826 10.1111/cas.13764 PMC6172072

[15] Bhan A, Soleimani M, Mandal SS. Long noncoding RNA and cancer: a new paradigm. Cancer Res. 2017;77(15):3965–81. 28701486 10.1158/0008-5472.CAN-16-2634 PMC8330958

[16] Tacutu R, Thornton D, Johnson E, Budovsky A, Barardo D, Craig T, Diana E, Lehmann G, Toren D, Wang J, et al. Human ageing genomic resources: new and updated databases. Nucleic Acids Res. 2018;46(D1):D1083–90. 29121237 10.1093/nar/gkx1042 PMC5753192

[17] Ding Y, Li T, Li M, Tayier T, Zhang M, Chen L, Feng S. a novel autophagy-related lncRNA gene signature to improve the prognosis of patients with melanoma. Biomed Res Int. 2021;2021:8848227. 34250091 10.1155/2021/8848227 PMC8238568

[18] Kaur A, Webster MR, Marchbank K, Behera R, Ndoye A, Kugel CH 3rd, Dang VM, Appleton J, O’Connell MP, Cheng P, et al. sFRP2 in the aged microenvironment drives melanoma metastasis and therapy resistance. Nature. 2016;532(7598):250–4. 27042933 10.1038/nature17392 PMC4833579

[19] Huang R, Mao M, Lu Y, Yu Q, Liao L. A novel immune-related genes prognosis biomarker for melanoma: associated with tumor microenvironment. Aging (Albany NY). 2020;12(8):6966–80. 32310824 10.18632/aging.103054 PMC7202520

[20] Lim SY, Lee JH, Diefenbach RJ, Kefford RF, Rizos H. Liquid biomarkers in melanoma: detection and discovery. Mol Cancer. 2018;17(1):8. 29343260 10.1186/s12943-018-0757-5 PMC5772714

[21] Çakır U, Balogh P, Ferenczik A, Brodszky V, Krenács T, Kárpáti S, Sárdy M, Holló P, Fábián M. G protein-coupled estrogen receptor 1 and collagen XVII endodomain expression in human cutaneous melanomas: can they serve as prognostic factors? Pathol Oncol Res. 2024;30:1611809. 39252786 10.3389/pore.2024.1611809 PMC11381273

[22] Zeng S, Chen XI, Yi Q, Thakur A, Yang H, Yan Y, Liu S. CRABP2 regulates infiltration of cancer-associated fibroblasts and immune response in melanoma. Oncol Res. 2023;32(2):261–72. 38186580 10.32604/or.2023.042345 PMC10765133

[23] Kodali N, Alomary S, Bhattaru A, Eldaboush A, Schwartz RA, Lipner SR. Gender and melanoma subtype-based prognostic implications of MUC16 and TTN co-occurrent mutations in melanoma: a retrospective multi-study analysis. Cancer Med. 2024;13(17):e70199. 39240165 10.1002/cam4.70199 PMC11378355

[24] Rong D, Su Y, Jia D, Zeng Z, Yang Y, Wei D, Lu H, Cao Y. Experimentally validated oxidative stress -associated prognostic signatures describe the immune landscape and predict the drug response and prognosis of SKCM. Front Immunol. 2024;15:1387316. 38660305 10.3389/fimmu.2024.1387316 PMC11039952

[25] Slusher N, Jones N, Nonaka T. Liquid biopsy for diagnostic and prognostic evaluation of melanoma. Front Cell Dev Biol. 2024;12:1420360. 39156972 10.3389/fcell.2024.1420360 PMC11327088

[26] Lukacova E, Hanzlikova Z, Podlesnyi P, Sedlackova T, Szemes T, Grendar M, Samec M, Hurtova T, Malicherova B, Leskova K, et al. Novel liquid biopsy CNV biomarkers in malignant melanoma. Sci Rep. 2024;14(1):15786. 38982214 10.1038/s41598-024-65928-y PMC11233564

[27] Kött J, Hoehne IL, Heidrich I, Zimmermann N, Reese KL, Zell T, Geidel G, Rünger A, Schneider SW, Pantel K, et al. High serum levels of CCL20 are associated with recurrence and unfavorable overall survival in advanced melanoma patients receiving immunotherapy. Cancers (Basel). 2024. 10.3390/cancers16091737. 38730689 10.3390/cancers16091737 PMC11083498

[28] Andre M, Caobi A, Miles JS, Vashist A, Ruiz MA, Raymond AD. Diagnostic potential of exosomal extracellular vesicles in oncology. BMC Cancer. 2024;24(1):322. 38454346 10.1186/s12885-024-11819-4 PMC10921614

[29] Lin JX, Leonard WJ. The common cytokine receptor gamma chain family of cytokines. Cold Spring Harb Perspect Biol. 2018. 10.1101/cshperspect.a028449. 29038115 10.1101/cshperspect.a028449 PMC6120701

[30] Saeidian AH, Youssefian L, Huang CY, Palizban F, Naji M, Saffarian Z, Mahmoudi H, Goodarzi A, Sotoudeh S, Vahidnezhad F, et al. Whole-transcriptome sequencing-based concomitant detection of viral and human genetic determinants of cutaneous lesions. JCI Insight. 2022. 10.1172/jci.insight.156021. 35316210 10.1172/jci.insight.156021 PMC9089792

[31] Quintana E, Shackleton M, Sabel MS, Fullen DR, Johnson TM, Morrison SJ. Efficient tumour formation by single human melanoma cells. Nature. 2008;456(7222):593–8. 19052619 10.1038/nature07567 PMC2597380

[32] Li Z, Wei J, Zheng H, Zhang Y, Song M, Cao H, Jin Y. The new horizon of biomarker in melanoma patients: a study based on autophagy-related long non-coding RNA. Medicine (Baltimore). 2022;101(1):e28553. 35029926 10.1097/MD.0000000000028553 PMC8735716

[33] Chen P, Gao Y, Ouyang S, Wei L, Zhou M, You H, Wang Y. A Prognostic model based on immune-related long non-coding RNAs for patients with cervical cancer. Front Pharmacol. 2020;11:585255. 33328990 10.3389/fphar.2020.585255 PMC7734341

[34] Meng C, Zhou JQ, Liao YS. Autophagy-related long non-coding RNA signature for ovarian cancer. J Int Med Res. 2020;48(11):300060520970761. 33179541 10.1177/0300060520970761 PMC7673061

[35] Sun Z, Jing C, Xiao C, Li T. An autophagy-related long non-coding RNA prognostic signature accurately predicts survival outcomes in bladder urothelial carcinoma patients. Aging (Albany NY). 2020;12(15):15624–37. 32805727 10.18632/aging.103718 PMC7467376

[36] Zhou W, Xu S, Deng T, Zhou R, Wang C. LncRNA USP30-AS1 promotes the survival of acute myeloid leukemia cells by cis-regulating USP30 and ANKRD13A. Hum Cell. 2022;35(1):360–78. 34694569 10.1007/s13577-021-00636-7 PMC8732929

[37] Dai S, Li N, Zhou M, Yuan Y, Yue D, Li T, Zhang X. LncRNA EBLN3P promotes the progression of osteosarcoma through modifying the miR-224-5p/Rab10 signaling axis. Sci Rep. 2021;11(1):1992. 33479458 10.1038/s41598-021-81641-6 PMC7820338

[38] Li H, Wang M, Zhou H, Lu S, Zhang B. Long noncoding RNA EBLN3P promotes the progression of liver cancer via alteration of microRNA-144-3p/DOCK4 signal. Cancer Manag Res. 2020;12:9339–49. 33061623 10.2147/CMAR.S261976 PMC7532886

[39] Xu XH, Song W, Li JH, Huang ZQ, Liu YF, Bao Q, Shen ZW. Long non-coding RNA EBLN3P regulates UHMK1 expression by sponging miR-323a-3p and promotes colorectal cancer progression. Front Med (Lausanne). 2021;8:651600. 34109193 10.3389/fmed.2021.651600 PMC8180563

[40] Magrin L, Fanale D, Brando C, Fiorino A, Corsini LR, Sciacchitano R, Filorizzo C, Dimino A, Russo A, Bazan V. POLE, POLD1, and NTHL1: the last but not the least hereditary cancer-predisposing genes. Oncogene. 2021;40(40):5893–901. 34363023 10.1038/s41388-021-01984-2

[41] Tang H, You T, Sun Z, Bai C. A Comprehensive Prognostic Analysis of POLD1 in Hepatocellular Carcinoma. BMC Cancer. 2022;22(1):197. 35189839 10.1186/s12885-022-09284-y PMC8862270

[42] Sehested A, Meade J, Scheie D, Ostrup O, Bertelsen B, Misiakou MA, Sarosiek T, Kessler E, Melchior LC, Munch-Petersen HF, et al. Constitutional POLE variants causing a phenotype reminiscent of constitutional mismatch repair deficiency. Hum Mutat. 2022;43(1):85–96. 34816535 10.1002/humu.24299

[43] Wimmer K, Beilken A, Nustede R, Ripperger T, Lamottke B, Ure B, Steinmann D, Reineke-Plaass T, Lehmann U, Zschocke J, et al. A novel germline POLE mutation causes an early onset cancer prone syndrome mimicking constitutional mismatch repair deficiency. Fam Cancer. 2017;16(1):67–71. 27573199 10.1007/s10689-016-9925-1 PMC5243902

[44] Smirnov A, Anemona L, Novelli F, Piro CM, Annicchiarico-Petruzzelli M, Melino G, Candi E. p63 is a promising marker in the diagnosis of unusual skin cancer. Int J Mol Sci. 2019. 10.3390/ijms20225781. 31744230 10.3390/ijms20225781 PMC6888618

[45] Di Como CJ, Urist MJ, Babayan I, Drobnjak M, Hedvat CV, Teruya-Feldstein J, Pohar K, Hoos A, Cordon-Cardo C. p63 expression profiles in human normal and tumor tissues. Clin Cancer Res. 2002;8(2):494–501. 11839669

[46] Matin RN, Chikh A, Chong SL, Mesher D, Graf M, Sanza P, Senatore V, Scatolini M, Moretti F, Leigh IM, et al. p63 is an alternative p53 repressor in melanoma that confers chemoresistance and a poor prognosis. J Exp Med. 2013;210(3):581–603. 23420876 10.1084/jem.20121439 PMC3600906

[47] Ma X, Dong L, Liu X, Ou K, Yang L. POLE/POLD1 mutation and tumor immunotherapy. J Exp Clin Cancer Res. 2022;41(1):216. 35780178 10.1186/s13046-022-02422-1 PMC9250176

[48] Szinovacz ME, DeViney S, Atkinson MP. Effects of surrogate parenting on grandparents’ well-being. J Gerontol B Psychol Sci Soc Sci. 1999;54(6):S376-388. 10625973 10.1093/geronb/54b.6.s376

[49] Wang X, Yue Y, Tan J, Kou F, Su B, Xie J, Yan S. LncRNA EBLN3P accelerates cell proliferation and invasion of colon cancer through modulating the miR-519d-3p/ZFP91 Axis. Cancer Biother Radiopharm. 2024. 10.1089/cbr.2022.0089. 38597324 10.1089/cbr.2022.0089

[50] Bhoumik A, Ronai Z. ATF2: a transcription factor that elicits oncogenic or tumor suppressor activities. Cell Cycle. 2008;7(15):2341–5. 18677098 10.4161/cc.6388

[51] Bhoumik A, Fichtman B, Derossi C, Breitwieser W, Kluger HM, Davis S, Subtil A, Meltzer P, Krajewski S, Jones N, et al. Suppressor role of activating transcription factor 2 (ATF2) in skin cancer. Proc Natl Acad Sci U S A. 2008;105(5):1674–9. 18227516 10.1073/pnas.0706057105 PMC2234203

[52] Chen M, Liu Y, Yang Y, Qiu Y, Wang Z, Li X, Zhang W. Emerging roles of activating transcription factor (ATF) family members in tumourigenesis and immunity: Implications in cancer immunotherapy. Genes Dis. 2022;9(4):981–99. 35685455 10.1016/j.gendis.2021.04.008 PMC9170601

[53] Isotalo T, Talja M, Tammela TL, Törmälä P, Paasimaa S, Andersson L. Cytotoxicity testing of a new caprolactone-coated self-expanding bioabsorbable self-reinforced poly-L-lactic acid urethral stent. Urol Res. 1999;27(2):149–52. 10424397 10.1007/s002400050101

[54] Minato H, Nakanuma Y, Terada T. Expression of blood group-related antigens in cholangiocarcinoma in relation to non-neoplastic bile ducts. Histopathology. 1996;28(5):411–9. 8735716 10.1046/j.1365-2559.1996.343384.x

[55] Claus JJ, van Gool WA, Teunisse S, Walstra GJ, Kwa VI, Hijdra A, Verbeeten B Jr, Koelman JH, Bour LJ. Ongerboer De Visser BW: predicting survival in patients with early Alzheimer’s disease. Dement Geriatr Cogn Disord. 1998;9(5):284–93. 9701680 10.1159/000017073

[56] Zheng X, Huang C, Lin Y, Han B, Chen Y, Li C, Li J, Ding Y, Song X, Wang W, et al. Generation of inactivated IL2RG and RAG1 monkeys with severe combined immunodeficiency using base editing. Signal Transduct Target Ther. 2023;8(1):327. 37661226 10.1038/s41392-023-01544-y PMC10475462

[57] Noto FK, Sangodkar J, Adedeji BT, Moody S, McClain CB, Tong M, Ostertag E, Crawford J, Gao X, Hurst L, et al. The SRG rat, a sprague-dawley Rag2/Il2rg double-knockout validated for human tumor oncology studies. PLoS ONE. 2020;15(10):e0240169. 33027304 10.1371/journal.pone.0240169 PMC7540894

[58] Yue Y, Cai X, Lu C, Sechi LA, Solla P, Li S. Unraveling the prognostic significance and molecular characteristics of tumor-infiltrating B lymphocytes in clear cell renal cell carcinoma through a comprehensive bioinformatics analysis. Front Immunol. 2023;14:1238312. 37908350 10.3389/fimmu.2023.1238312 PMC10613680

[59] Li LY, Yang Q, Jiang YY, Yang W, Jiang Y, Li X, Hazawa M, Zhou B, Huang GW, Xu XE, et al. Interplay and cooperation between SREBF1 and master transcription factors regulate lipid metabolism and tumor-promoting pathways in squamous cancer. Nat Commun. 2021;12(1):4362. 34272396 10.1038/s41467-021-24656-x PMC8285542

[60] Parsons JK, Saria EA, Nakayama M, Vessella RL, Sawyers CL, Isaacs WB, Faith DA, Bova GS, Samathanam CA, Mitchell R, et al. Comprehensive mutational analysis and mRNA isoform quantification of TP63 in normal and neoplastic human prostate cells. Prostate. 2009;69(5):559–69. 19142959 10.1002/pros.20904 PMC2875878

[61] Han J, Hu Y, Ding S, Liu S, Wang H. The analysis of the pyroptosis-related genes and hub gene TP63 ceRNA axis in osteosarcoma. Front Immunol. 2022;13:974916. 36389801 10.3389/fimmu.2022.974916 PMC9664215

[62] Enguita FJ, Leitão AL, McDonald JT, Zaksas V, Das S, Galeano D, Taylor D, Wurtele ES, Saravia-Butler A, Baylin SB, et al. The interplay between lncRNAs, RNA-binding proteins and viral genome during SARS-CoV-2 infection reveals strong connections with regulatory events involved in RNA metabolism and immune response. Theranostics. 2022;12(8):3946–62. 35664076 10.7150/thno.73268 PMC9131284

[63] Albert TJ, Lamb D, Piazza MR, Flanders AE, Balderston RA, Cotler JM. MRI evaluation of fusion mass incorporation after anterior cervical bony fusions: preliminary findings. Paraplegia. 1993;31(10):667–74. 8259330 10.1038/sc.1993.107

[64] Chen M, Chi Y, Chen H, Zhao L. Long non-coding RNA USP30-AS1 aggravates the malignant progression of cervical cancer by sequestering microRNA-299-3p and thereby overexpressing PTP4A1. Oncol Lett. 2021;22(1):505. 33986866 10.3892/ol.2021.12766 PMC8114562

[65] Ge X, Lei S, Wang P, Wang W, Wang W. The metabolism-related lncRNA signature predicts the prognosis of breast cancer patients. Sci Rep. 2024;14(1):3500. 38347041 10.1038/s41598-024-53716-7 PMC10861477

[66] Xu H, Lu M, Liu Y, Ren F, Zhu L. Identification of a pyroptosis-related long non-coding RNA Signature for prognosis and its related ceRNA regulatory network of ovarian cancer. J Cancer. 2023;14(16):3151–68. 37859811 10.7150/jca.88485 PMC10583579

[67] Wan J, Guo C, Fang H, Xu Z, Hu Y, Luo Y. Autophagy-related long non-coding rna is a prognostic indicator for bladder cancer. Front Oncol. 2021;11:647236. 33869042 10.3389/fonc.2021.647236 PMC8049181

[68] Zhang M, Yang L, Wang Y, Zuo Y, Chen D, Guo X. Comprehensive prediction of immune microenvironment and hot and cold tumor differentiation in cutaneous melanoma based on necroptosis-related lncRNA. Sci Rep. 2023;13(1):7299. 37147395 10.1038/s41598-023-34238-0 PMC10163022

[69] Kinghorn KJ, Castillo-Quan JI, Bartolome F, Angelova PR, Li L, Pope S, Cocheme HM, Khan S, Asghari S, Bhatia KP, et al. Loss of PLA2G6 leads to elevated mitochondrial lipid peroxidation and mitochondrial dysfunction. Brain. 2015;138(Pt 7):1801–16. 26001724 10.1093/brain/awv132 PMC4559908

[70] Luo M, Wu L, Zhang K, Wang H, Zhang T, Gutierrez L, O’Connell D, Zhang P, Li Y, Gao T, et al. miR-137 regulates ferroptosis by targeting glutamine transporter SLC1A5 in melanoma. Cell Death Differ. 2018;25(8):1457–72. 29348676 10.1038/s41418-017-0053-8 PMC6113319

[71] Pappalardo F, Russo G, Candido S, Pennisi M, Cavalieri S, Motta S, McCubrey JA, Nicoletti F, Libra M. Computational modeling of PI3K/AKT and MAPK signaling pathways in melanoma cancer. PLoS ONE. 2016;11(3):e0152104. 27015094 10.1371/journal.pone.0152104 PMC4807832

[72] Schlegel J, Sambade MJ, Sather S, Moschos SJ, Tan AC, Winges A, DeRyckere D, Carson CC, Trembath DG, Tentler JJ, et al. MERTK receptor tyrosine kinase is a therapeutic target in melanoma. J Clin Invest. 2013;123(5):2257–67. 23585477 10.1172/JCI67816 PMC3639697

[73] Flaherty KT, Infante JR, Daud A, Gonzalez R, Kefford RF, Sosman J, Hamid O, Schuchter L, Cebon J, Ibrahim N, et al. Combined BRAF and MEK inhibition in melanoma with BRAF V600 mutations. N Engl J Med. 2012;367(18):1694–703. 23020132 10.1056/NEJMoa1210093 PMC3549295

[74] Ribas A, Puzanov I, Dummer R, Schadendorf D, Hamid O, Robert C, Hodi FS, Schachter J, Pavlick AC, Lewis KD, et al. Pembrolizumab versus investigator-choice chemotherapy for ipilimumab-refractory melanoma (KEYNOTE-002): a randomised, controlled, phase 2 trial. Lancet Oncol. 2015;16(8):908–18. 26115796 10.1016/S1470-2045(15)00083-2 PMC9004487

